# Long non-coding RNA GAS5 suppresses pancreatic cancer metastasis through modulating miR-32-5p/PTEN axis

**DOI:** 10.1186/s13578-017-0192-0

**Published:** 2017-12-04

**Authors:** Zhi-Qiang Gao, Jun-feng Wang, De-Hua Chen, Xue-Song Ma, Yang Wu, Zhe Tang, Xiao-Wei Dang

**Affiliations:** Department of Hepatobiliary and Pancreatic Surgery, The First Affiliated Hospital of Zhengzhou University, No. 1 Jianshe East Road, Zhengzhou, Henan People’s Republic of China

**Keywords:** Pancreatic cancer, GAS5, miR-32-5p, PTEN

## Abstract

**Background:**

Long non-coding RNA growth arrest-specific transcript 5 (lncRNA GAS5) is a well-known tumor suppressor in the pathogenesis of a variety of human cancers. The precise role of GAS5 in pancreatic cancer (PC) progression is currently unknown, so the aim of this study was to explore the functional participation of GAS5 in PC metastasis.

**Methods:**

The expression changes of GAS5, miR-32-5p and PTEN in human PC specimens and cell lines were compared by means of molecular biology methods. Transfection of the recombinant plasmid was applied to modulate the expression levels of the target genes. RIP and RNA pull-down assays were designed to investigate the interaction between GAS5 and miR-32-5p. The effect of GAS5 and miR-32-5p on PC progression was assessed with cell proliferation, migration, invasion and apoptosis in vitro.

**Results:**

GAS5 and PTEN protein were decreased in human PC tissues and cells, but miR-32-5p was increased. GAS5 induction greatly inhibited the proliferation, migration and invasion of PC cells PANC-1 and BxPC-3 in vitro and simultaneously induced cell apoptosis. Moreover, GAS5 positively regulated the expression of PTEN through miR-32-5p. Furthermore, GAS5 suppressed the proliferation, migration and invasion of PC cells through regulating miR-32-5p/PTEN axis. Additionally, this finding was further supported by the results of in vivo experiments.

**Conclusion:**

GAS5 could positively regulate PTEN-induced tumor-suppressor pathway via miR-32-5p, thereby suppressing PC metastasis.

**Electronic supplementary material:**

The online version of this article (10.1186/s13578-017-0192-0) contains supplementary material, which is available to authorized users.

## Background

Pancreatic cancer (PC) is a malignant neoplasm in digestive tract with a high degree of malignancy, which is difficult to diagnose and treat [1]. About 90% are ductal adenocarcinoma derived from glandular epithelium and prognosis is extremely poor [2]. The early diagnostic accuracy rate of PC is low, but the operative mortality is high because of the high recurrence rate.

Long noncoding RNA (lncRNA) is a non-coding RNA transcript with length greater than 200 nucleotides, and plays an important regulatory role in tumor biological processes such as growth and metastasis [3]. LncRNA growth arrest-specific transcript 5 (GAS5) has been identified as one of the important regulatory factor in the pathogenesis of a variety of human cancers, including PC. The low expression of GAS5 was positively related to the shortening of the overall survival period of cancer patients with colorectal cancer and thyroid cancer [4, 5]. GAS5, acts as a tumor suppressor, has been shown to be extensively involved in the proliferation, apoptosis, migration and invasion of tumor cells [6]. For instance, GAS5 inhibited the proliferation, migration and invasion of human glioma cells in vitro and in mice via promoting tumor suppressor Bcl-2-modifying factor (bmf) and Plexin C1 expression [7]. More recently, GAS5 has been reported to down-regulate in human PC tissues, and GAS5 overexpression significantly inhibited the proliferation of PC cells in vitro, suggesting the important role of GAS5 in PC context [8]. However, its specific mechanism still needs further study and the relevant research is very limited. Many studies have shown that GAS5 induced inhibitory effect on the migration and invasion of different types of tumor cells in vitro and in vivo, including renal cell carcinoma, lung cancer, hepatocellular carcinoma, ovarian cancer, cervical cancer [6, 9–11]. The potential role of GAS5 in PC metastasis is currently unknown.

MicroRNA (miRNA) is an important class of small ncRNA that induces the translation inhibition and degradation of target mRNA through targeting the mRNA 3-untranslated region (3′-UTR) [12]. MiR-32-5p is an important mediator that is closely related to cancer-specific survival of bladder tumors [13]. MiR-32-5p was down-regulated in blood from prostate cancer patients, and thus was expected to be a new indicator for prostate cancer diagnosis [14]. Wu et al. found that miR-32 down-regulated anti-oncogene phosphatase and tensin homologue (PTEN), thereby contributing to the migration and invasion of colorectal carcinoma cells [15]. PTEN is also a negative regulator of PC progression. PTEN was decreased in PC and PTEN-induced PI3K/Akt signaling inactivation impeded the migration, invasion and growth of PC [16]. Recent advances in drug resistance revealed that PTEN was related to 5-fluorouracil resistance of PC cells and overexpressed PTEN could rescue 5-fluorouracil resistance in PC cells [17]. Obviously, PTEN exerts important tumor-suppressive roles in PC progression. In view of the findings, we were interested in that whether miR-32-5p promoted PC metastasis via modulating PTEN level.

Numerous of evidence implicated that GAS5 regulated the expression of genes and cell signaling involved in cell cycle control and cellar metabolism through interaction with miRNAs [18]. MiR-21, miR-103, miR-222, were identified as the downstream targets of GAS5 [7, 19, 20]. The bioinformatics analysis (online systems) showed that the binding site of GAS5 also existed in miR-32-5p, suggesting a potential interaction between GAS5 and miR-32-5p. We therefore inferred that GAS5 was involved in PC metastasis through modulating miR-32-5p/PTEN axis.

## Methods

### Human PC tissues collection

All protocols were supported by the ethics committee of the First Affiliated Hospital of Zhengzhou University, and the informed consents obtained from all patients recruited in this study prior surgery. A total of 22 patients with pancreatic ductal adenocarcinomas diagnosed by pathological review were recruited in the study, and human PC specimens (n = 22) and their para-carcinoma tissues (n = 22) were collected from PC patients underwent cancer resection at the First Affiliated Hospital of Zhengzhou University. All patients were not treated with chemotherapy, Chinese medicine or radiotherapy before surgical resection. The characteristics of the patients were included in Additional file 1: Table S1. The collected tissues were placed in − 80 °C refrigerator until detection.

### Cell culture

Human pancreatic ductal adenocarcinoma cell lines PANC-1 and BxPC-3, and normal human pancreatic duct epithelial cells HPDE6-C7 were purchased from American Type Culture Collection (ATCC, USA). PANC-1 cells were cultured in DMEM medium supplemented with 10% fetal bovine serum (FBS, Gibco) and 1% penicillin–streptomycin (HyClone) with 5% CO_2_ at 37 °C. BxPC-3 cells were maintained in 1640-RPMI medium containing 10% FBS (Gibco) and 1% penicillin–streptomycin (HyClone) with 5% CO_2_ at 37 °C. HPDE6-C7 cells were cultured in keratinocyte serum free medium (K-SFM) containing bovine pituitary extract (5 mg/100 ml) and epidermal growth factor (EGF, 0.5 µg/100 ml, Invitrogen) with 5% CO_2_ at 37 °C.

### Quantitative real-time PCR (qRT-PCR)

Total RNA was extracted from the tissues or cells using TRIzol reagent (Invitrogen) according to the manufacturer’s instructions. Total RNA was reverse transcribed into cDNA using a miScript II RT Kit (Qiagen) for analysis of miR-32-5p expression, and the total RNA was applied in a cDNA Reverse Transcription Kit (Applied Biosystems) for detection of GAS5 and PTEN mRNA. The relative expression of miR-32-5p was determined by using SYBR Green-based miScript PCR Array (Qiagen) according to the manufacturer’s instructions and normalized to U6. The relative expression of GAS5 and PTEN mRNA was detected using SYBR Premix Ex Taq II (TaKaRa) and normalized to GAPDH. The specific primers for miR-32-5p (forward 5′-3′: CGGTATTGCACATTACTAAGTTGCA; reverse 5′-3′: CTCGCTTCGGCAGCACA), GAS5 (forward 5′-3′: AAGCCATTGGCACACAGGCATTAG; reverse 5′-3′: AGAACCATTAAGCTGGTCCAGGCA), PTEN (forward 5′-3′: ACCAGTGGCACTGTTGTTTCAC; reverse 5′-3′: TTCCTCTGGTCCTGGTATGAAG), U6 (forward 5′-3′: CTCGCTTCGGCAGCACA; reverse 5′-3′: AACGCTTCACGAATTTGCGT) and GAPDH (forward 5′-3′: ACAACTTTGGTATCGTGGAAGG; reverse 5′-3′: GCCATCACGCCACAGTTTC) were provided by Sangon Biotech (Shanghai) Co., Ltd. (China).

### Western blotting

Western blotting was performed to detect the expression of PTEN protein in the tissues or cells. The cells were lysed in lysis buffer (Thermo Scientific) and then the protein was collected by centrifugation at 12,000 rpm for 10 min. The total protein of each sample was quantified by BCA Protein Assay Kit (Beyotime Biotechnology, No. P0011). Proteins were separated by SDS-PAGE and then were electroblotted to PVDF membrane (Millipore). The membrane was blocked with 5% skim milk in TBST for 1 h and the protein on membrane was then incubated with the specific primary antibody for PTEN (1:500; Cell Signaling Technology) or β-actin (1:1000; Abcam) overnight. The proteins were further incubated with the appropriate horseradish peroxidase-conjugated secondary antibody for 1 h. The protein bands were visualized using ECL chemiluminescence system (Thermo Fisher Scientific). β-actin was used as the control for PTEN protein.

### Cell Counting Kit-8 assay

The relative cell viability was evaluated by Cell Counting Kit-8 assay, which is a widely used method for analysis of cell viability. In brief, PANC-1 and BxPC-3 cells were cultured in 96-well plates with 6 × 10^3^ cells/well. CCK-8 solution (10 μl, Dojindo) was added in each well and incubated with the cells for 1 h. The absorbance at 450 nm was measured with a microplate reader (Bio-Rad). Each cell group duplicated into six wells and the experiment was repeated in triplicate.

### Detection of migration and invasion with Transwell system

The transwell system (polycarbonate with 8 μm pore) (Corning) was used to measure the migration and invasion of PANC-1 and BxPC-3 cells. For migration, 2 × 10^4^ cells cultured in serum-free medium were placed in the chambers without Matrigel. For invasion, 2 × 10^5^ cells cultured in serum-free medium were placed in the chambers with Matrigel. The medium containing 10% FBS was added in the lower chamber. After incubation for 24 h at 37 °C with 5% CO_2_, the cells in the upper chamber were scraped off by medical cotton stickers, and the cells on the other side of the membrane were fixed by methanol and stained with 0.5% crystal violet for 2 h. The number of stained cells was counted on an inverted microscope with five random fields.

### Flow cytometry analysis of apoptosis

After transfection, the apoptosis level of PANC-1 and BxPC-3 cells were analyzed using flow cytometry. The cells were collected and washed two times with PBS. Subsequently, the cells were stained by Annexin V-FITC and PI dye for 15 min in dark according to the manufacturer’s instructions. The stained cells were analyzed by flow cytometry (BD Biosciences) and were counted using the CellQuest software (BD Biosciences). This experiment was repeated three times.

### Cell transfection

The pcDNA vector carrying GAS5 (pcDNA-GAS5) was constructed at Shanghai Genechem Co., Ltd. (China) to induce GAS5 in PC cells. The specific interference sequence (5-CTTGCCTGGACCAGCTTAATT-3) for GAS5 (siRNA-GAS5) was used to down-regulate the expression of GAS5. The commercial miR-32-5p inhibitor and mimic were purchased from GenePharma (China). PANC-1 and BxPC-3 cells were cultured in 24-well plates and transfected with pcDNA-GAS5 (2 µg) or siRNA-GAS5 or miR-32-5p inhibitor (150 nM) or mimic (100 nM) or the appropriate negative control using Lipofectamine 2000 (Invitrogen), according to the manufacturer’s instructions.

### RNA immunoprecipitation (RIP) assay

The RIP assay was performed to explore the interaction between GAS5 and miR-32-5p by using EZ-Magna RIP RNA-binding protein immunoprecipitation kit (Millipore). PANC-1 cells were lysed, and the cell lysis was then incubated with anti-human Ago2 antibodies (Millipore) coated on magnetic beads in RIP buffer. Input and normal IgG were used as controls. The precipitated RNAs were isolated and reverse transcribed in cDNA to analyze GAS5 and miR-32-5p level using qRT-PCR.

### RNA pull-down assay

GAS5 RNA was transcribed using T7 RNA polymerase (Roche) and biotin-labeled by using Biotin RNA Labeling Mix (Roche). The RNA pull-down assay was performed with the Magnetic RNA–Protein Pull-Down Kit (Thermo Fisher) according to the manufacturer’s instructions. Biotinylated GAS5 was incubated with streptavidin beads from kit at 4 °C overnight. The cell lysate was added, then hatching for 4 h sequentially at 4 °C. The beads were washed for three times. The level of Ago2 protein in the eluted complex was analyzed by western blotting, and the miR-32-5p level was determined by qRT-PCR according to the standard procedures.

### Animal study

Twenty-four female athymic BALB/c nude mice (4–5 week old) were provided by the animal center of Zhengzhou University. The animal study was supported by the ethics committee of the First Affiliated Hospital of Zhengzhou University, and all protocols were performed under the guidelines of the experimental animal management of the First Affiliated Hospital of Zhengzhou University.

PANC-1 cells were transfected with pcDNA-GAS5 or pcDNA, the negative control for pcDNA-GAS5. The normal control or negative control or PANC-1 cells (5 × 10^6^ cells) expressing GAS5 were used to induce subcutaneous tumors of mice (n = 6 in each group) by subcutaneous injection. The tumor volume was monitored weekly for 4 weeks and calculated using the formula, V (mm^3^) = 0.5 × L × W^2^ (V, volume; L, length; W, width). After the last measurement, the mice were executed, and all tumor tissues were dissected from the mice for analysis of the expression level of miR-32-5p and PTEN.

### Statistical analysis

All data from at least three independent experiments were represented as the mean ± standard deviation (SD). Statistical analysis was performed using the SPSS 19.0 software (USA). Difference analysis was conducted by Bonferroni t test. P < 0.05 was considered significant.

## Results

### GAS5 and PTEN protein were decreased in human PC tissues and cells, but miR-32-5p was increased

**Fig. 1:**
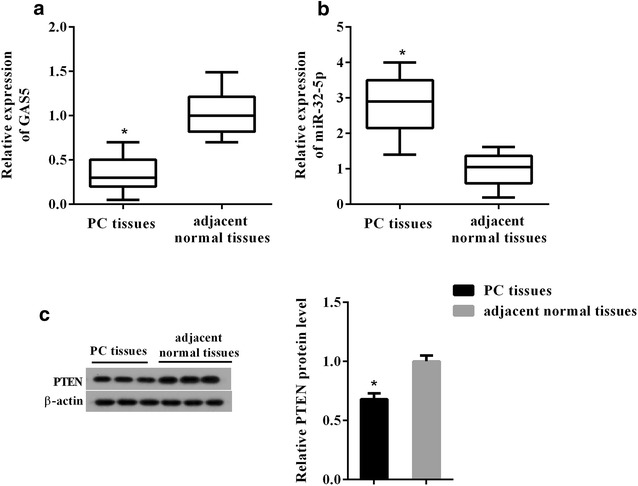
The expression profile of GAS5, miR-32-5p and PTEN protein in pancreatic cancer (PC) tissue. **a** The relative expression of GAS5 as determined by qRT-PCR in human PC tissues (n = 22). **b** qRT-PCR analysis of the relative expression of miR-32-5p in human PC tissues (n = 22). **c** The expression changes of PTEN protein in human PC tissues (n = 22). The adjacent normal tissues (n = 22) were used as control. ^*^P < 0.05 vs. adjacent normal tissues

A total of 22 human PC tissues and the adjacent normal tissues were collected for analysis of the expression profile of GAS5, miR-32-5p and PTEN protein in PC. The relative expression of GAS5 as determined by qRT-PCR in human PC tissues (n = 22) was obviously lower than that in normal tissues (n = 22) (Fig. 1a). Meanwhile, miR-32-5p expression was dramatically increased in PC tissues (n = 22) compared with the normal tissues (n = 22) (Fig. 1b). And a significant decrease in PTEN protein level was observed in PC tissues (n = 22) (Fig. 1c).

**Fig. 2:**
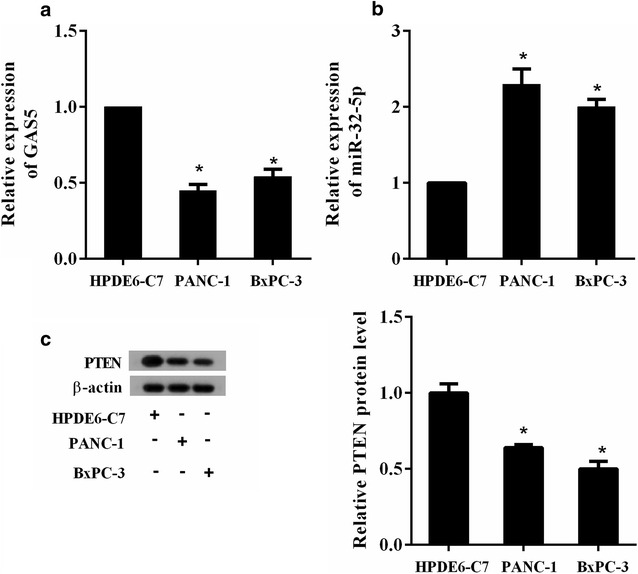
The expression profile of GAS5, miR-32-5p and PTEN protein in PC cell lines. qRT-PCR analysis of the relative expression of **a** GAS5 and **b** miR-32-5p in PC cell lines PANC-1 and BxPC-3, and normal human pancreatic duct epithelial cells HPDE6-C7. **c** The expression of PTEN protein in PANC-1, BxPC-3 and HPDE6-C7. ^*^P < 0.05 vs. HPDE6-C7

The expression changes of GAS5, miR-32-5p and PTEN protein in PC cells (PANC-1, BxPC-3) and normal human pancreatic duct epithelial cells HPDE6-C7 were also analyzed and compared. As shown in Fig. 2a, GAS5 had a relatively low expression both in PANC-1 and BxPC-3 cells. Whereas miR-32-5p was highly expressed in PC cells PANC-1 and BxPC-3 (Fig. 2b). The level of PTEN protein was markedly lower in PC cells than that in HPDE6-C7 cells (Fig. 2c).

### GAS5 inhibited the viability, migration and invasion of PC cells in vitro

**Fig. 3:**
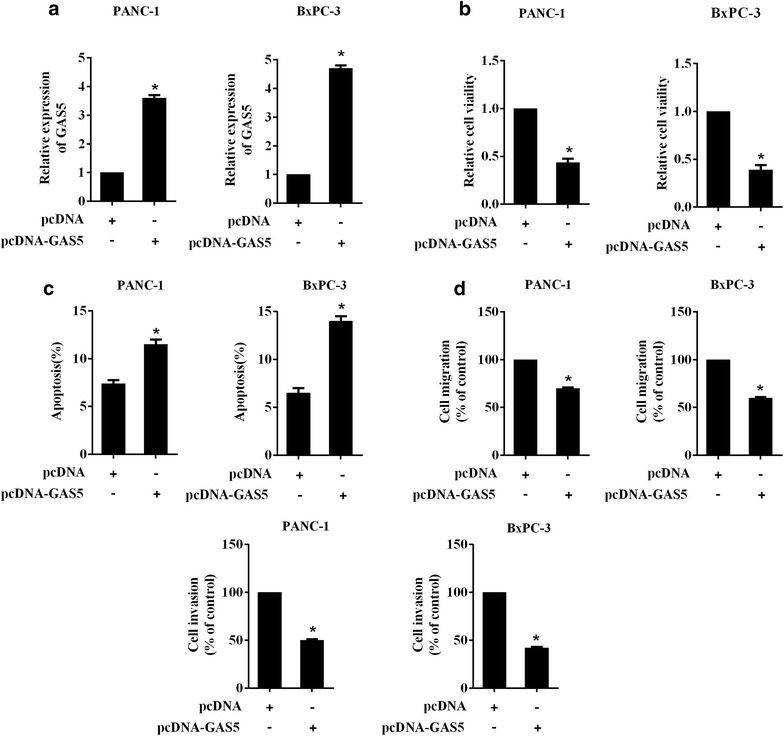
GAS5 inhibited the viability, migration and invasion of PC cells. The expression plasmid carrying GAS5 (pcDNA-GAS5) was transfected into PC cells PANC-1 and BxPC-3, empty pcDNA vector as control. **a** The expression of intracellular GAS5 as determined by qRT-PCR was increased by pcDNA-GAS5. **b** The detection of the relative cell viability of PC cells transfected with pcDNA-GAS5. **c** The apoptosis of PC cells was also assessed by flow cytometry. **d** The effect of GAS5 overexpression on the migration and invasion of PC cells in vitro. ^*^P < 0.05 vs. pcDNA

To explore the role of GAS5 in PC cells, GAS5 was induced in PANC-1 and BxPC-3 cells through transfection of pcDNA-GAS5 (Fig. 3a). The relative cell viability of PANC-1 and BxPC-3 cells transfected with pcDNA-GAS5 were significantly reduced compared with the cells transfected with the negative control for pcDNA-GAS5 (Fig. 3b). Moreover, the apoptosis percentage of PANC-1 and BxPC-3 cells was also significantly increased by pcDNA-GAS5 (Fig. 3c). Compared with the control plasmid, pcDNA-GAS5 inhibited the migration and invasion of PANC-1 and BxPC-3 cells (Fig. 3d). These data suggested that GAS5 could inhibit the proliferation, migration and invasion of PC cells in vitro.

### The interaction between GAS5 and miR-32-5p in PC cells

**Fig. 4:**
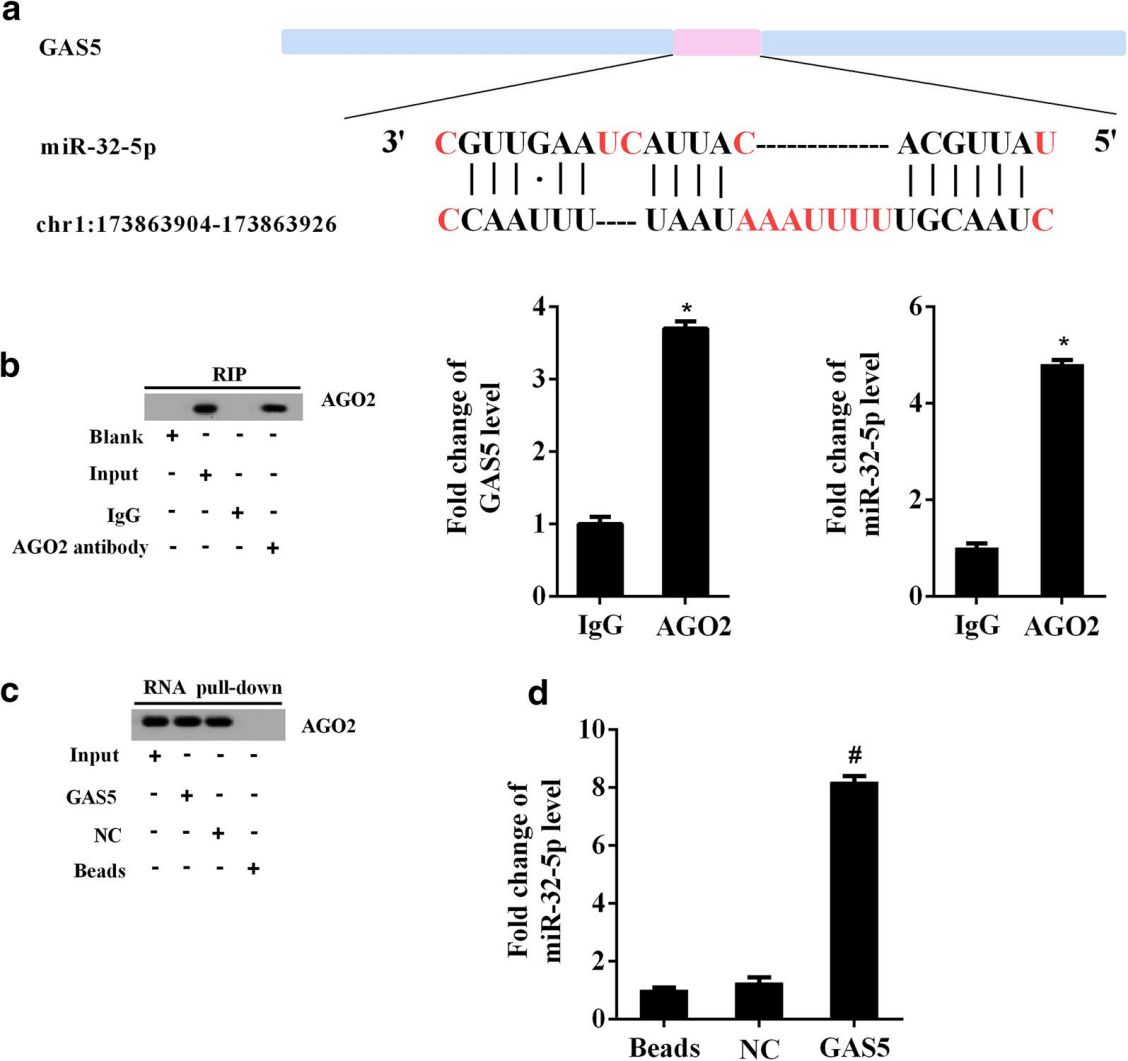
The interaction between GAS5 and miR-32-5p. **a** The putative binding sites between GAS5 and miR-32-5p. **b** RNA immunoprecipitation (RIP) assay was performed to observe the position relation between GAS5 and miR-32-5p. **c** RNA pull-down assay was used to further explore the interaction between GAS5 and miR-32-5p. ^*^P < 0.05 vs. IgG; ^#^P < 0.05 vs. NC

The bioinformatics analysis revealed potential combination of GAS5 and miR-32-5p, the putative binding sites as shown in Fig. 4a. In a further RIP experiment, GAS5 and miR-32-5p simultaneously existed in the production precipitated by anti-AGO2 (Fig. 4b). Consistent with this, RNA pull-down assay revealed that miR-32-5p could interact with GAS5 in PANC-1 cells possibly through a sequence-specific manner (Fig. 4c, d).

### GAS5 positively regulated the expression of PTEN through miR-32-5p

**Fig. 5:**
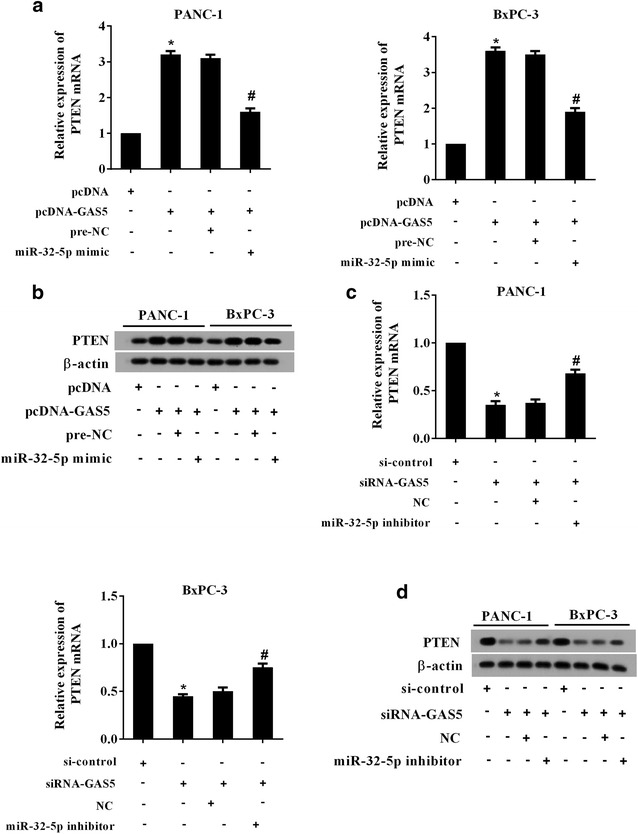
GAS5 positively regulated the expression of PTEN through miR-32-5p. **a**, **b** PC cells PANC-1 and BxPC-3 were transfected with pcDNA-GAS5 alone or with miR-32-5p mimic. The expression of PTEN was determined both at mRNA and protein level. ^*^P < 0.05 vs. pcDNA; ^#^P < 0.05 vs. pcDNA-GAS5 + pre-NC. **c**, **d** PC cells PANC-1 and BxPC-3 were transfected with si-GAS5 alone or with miR-32-5p inhibitor. The expression of PTEN both at mRNA and protein level was detected. ^*^P < 0.05 vs. si-control; ^#^P < 0.05 vs. si-GAS5 + NC

The expression trend of PTEN, a downstream target of miR-32-5p, was similar to GAS5 both in human PC tissues and cells. The co-transfection experiment was designed to investigate whether GAS5 could up-regulate the expression of PTEN by miR-32-5p. As shown in Fig. 5a, pcDNA-GAS5 significantly promoted the expression of PTEN mRNA in PANC-1 and BxPC-3 cells, but the level of PTEN mRNA aggressively reduced in PC cells transfected with pcDNA-GAS5 and miR-32-5p mimic. In addition, the protein level of PTEN was also elevated by GAS5 overexpression; however, the GAS5-induced increase in PTEN protein expression was suppressed markedly by miR-32-5p overexpression, suggesting that miR-32-5p negatively regulated the expression of PTEN and mediated the effect of GAS5 on PTEN expression (Fig. 5b). This finding was further confirmed by that miR-32-5p silencing combined with GAS5 down-regulation completely reversed the siRNA-GAS5-mediated inhibition on PTEN expression both at mRNA and protein levels (Fig. 5c, d). We further compared PTEN levels between miR-32-5p inhibitor plus si-control and miR-32-5p inhibitor plus si-GAS5. As shown in Additional file 2: Figure S1, compared with miR-32-5p inhibitor plus si-control, miR-32-5p inhibitor plus si-GAS5 had no significant influence on PTEN levels in PANC-1 and BxPC-3 cells.

### MiR-32-5p mediated the suppression of GAS5 on the viability, migration and invasion of PC cells

**Fig. 6:**
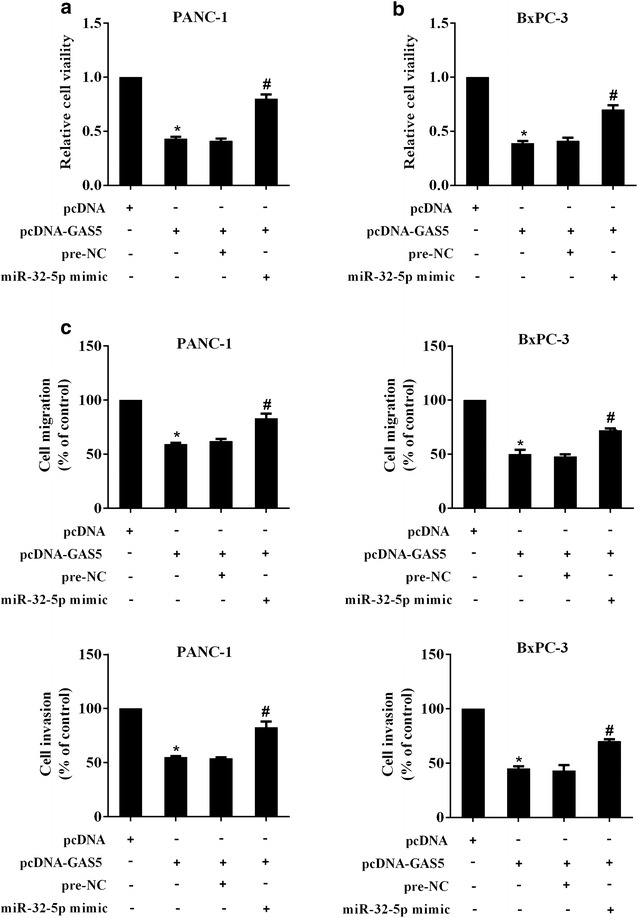
MiR-32-5p mediated the suppression of GAS5 on the viability, migration and invasion of PC cells. PC cells PANC-1 and BxPC-3 were divided into four groups: pcDNA, pcDNA-GAS5, pcDNA-GAS5 + pre-NC, pcDNA-GAS5 + miR-32-5p mimic. **a**, **b** The detection of the relative cell viability of PC cells treated as indicated. **c** The migration and invasion of PC cells was also assessed. ^*^P < 0.05 vs. pcDNA; ^#^P < 0.05 vs. pcDNA-GAS5 + pre-NC; ^&^P < 0.05 vs. pcDNA-GAS5 + miR-32-5p mimic

The co-transfection of the expression vector of GAS5 and miR-32-5p was designed to investigate the role of miR-32-5p in the GAS5-induced growth inhibition of PC cells. PANC-1 and BxPC-3 cells were transfected with pcDNA or pcDNA-GAS5 or pcDNA-GAS5 + pre-NC or pcDNA-GAS5 + miR-32-5p mimic. Clearly, over-expressed GAS5 alone significantly inhibited the viability, migration and invasion of both PANC-1 and BxPC-3 cells (Fig. 6a–c). Meanwhile, simultaneously overexpressing miR-32-5p and GAS5 greatly reversed the GAS5-induced repression on PC cells (Fig. 6a–c). PANC-1 and BxPC-3 cells were transfected with miR-32-5p inhibitor plus si-control or miR-32-5p inhibitor plus si-GAS5. It was found that miR-32-5p inhibitor plus si-GAS5 had no significant influence on the viability, migration and invasion of PANC-1 and BxPC-3 cells compared with miR-32-5p inhibitor plus si-control (Additional file 3: Figure S2). These findings collectively suggested that miR-32-5p mediated the effect of GAS5 on the viability, migration and invasion of PC cells.

### GAS5 overexpression inhibited PC cells tumorigenesis in vivo

**Fig. 7:**
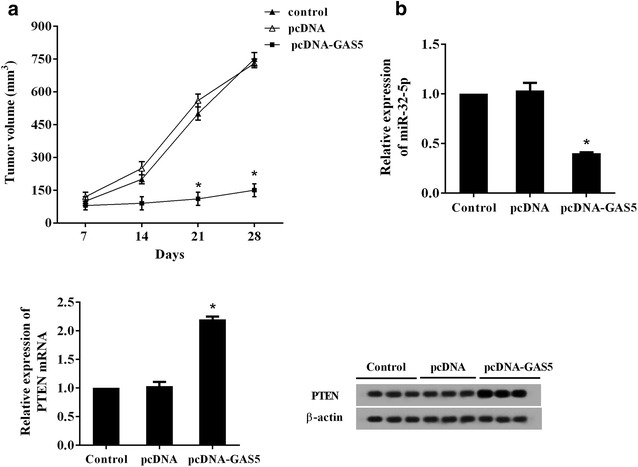
GAS5 overexpression inhibited PC cells tumorigenesis in vivo. PANC-1 cells were transfected with pcDNA or pcDNA-GAS5 and then 5 × 10^6^ cells suspended in 0.1 ml of PBS were injected in the posterior flank of the mice. **a** After a week of injection, the tumor volume of each mouse was monitored weekly for 4 weeks. **b** The expression of miR-32-5p and PTEN in tumor tissue induced by PANC-1 cells was detected after 28 days observation. ^*^P < 0.05 vs. pcDNA

PANC-1 cells were transfected with pcDNA or pcDNA-GAS5 and then 5 × 10^6^ cells suspended in 0.1 ml of PBS were injected in the posterior flank of the mice. After a week of injection, the tumor volume of each mouse was monitored weekly for 4 weeks and the results revealed that the tumorigenesis of the pcDNA-GAS5 transfected cells in nude mice were significantly reduced compared with the controls, as shown by the smaller volume and slow growth rate of tumor (Fig. 7a). The expressions of miR-32-5p and PTEN in tumor tissue were detected after 28 days observation. The expression of miR-32-5p was markedly inhibited in GAS5-over-expressed tumor tissue, whereas both PTEN mRNA and protein levels were increased (Fig. 7b).

## Discussion

Recent years, non-coding RNAs including lncRNAs and miRNAs are widely noticed due to their implication in tumorigenesis or metabolic disorders [21]. The non-coding RNAs, as the upstream regulators of cellular functional proteins and cell signaling, regulate the critical biological events in cancer biology. Specially, lncRNA growth arrest-specific transcript 5 (GAS5), although has only recently been identified, its role in human cancers has been concerned and studied greatly. Most studies hold that, GAS5 inhibited the occurrence and progression of human tumors [6, 22]. In this study, we found that GAS5 reduced both in human PC specimens (n = 22) and PC cell lines, suggesting a certain regulatory role of GAS5 dysregulation in PC context. Lu et al. reported that GAS5 was down-regulated in PC tissues (n = 23) and over-expressed GAS5 significantly inhibited the proliferation of PC cells in vitro [8]. Here, we also observed that the induction of GAS5 inhibited the proliferation, migration and invasion of PC cells and simultaneously induced cell apoptosis in vitro. GAS5 has been suggested to inhibit the migration and invasion of many types of tumor cells, but the inhibitory effect of GAS5 on PC cell migration and invasion was first reported in this work. Based on Lu et al. studies [8], our results provided strong evidence that GAS5 acted as tumor suppressor in PC pathogenesis.

To date, miR-32-5p was also reported to abnormally expressed in tumors and played important regulative roles [14]. Our data revealed that miR-32-5p was highly expressed in human PC tissues and cell lines, indicating that miR-32-5p may contribute to the development of PC. MiR-32 has been confirmed to promote the proliferation and migration of breast cancer cells, but the specific role of miR-32-5p in PC remains unknown [23]. MiR-32-5p was significantly decreased in fatty acids-treated human colorectal adenocarcinoma cells accompanied by a decrease in BCL-2 and BCL2L11 expression [24]. In addition, photodynamic therapy strongly down-regulated the expression miR-32-5p in oral cancer cells, but its roles in the therapeutic effect of photodynamic therapy are unknown [25]. Our results firstly identified that miR-32-5p was increased in PC tissues and cells, and was associated with the migration and invasion of PC cells.

LncRNAs can regulate the biological activity of miRNA, which is one of the important mechanisms of lncRNAs modulating cell physiological activities [26]. Elevated levels of GAS5 decreased the expression of miR-222 through directly targeting in glioma cells; meanwhile, tumor suppressor Bcl-2-modifying factor (bmf), as the downstream target of miR-222, was up-regulated, and thus inhibited glioma cell proliferation [7]. Combined with the results of bioinformatic analysis, we inferred that GAS5 may regulate the downstream molecules and signals of miR-32-5p by interacting with miR-32-5p that participates in the pathogenesis of PC. As expected, GAS5 negatively regulated miR-32-5p expression, thereby promoting the expression of phosphatase and tensin homologue (PTEN), which is the confirmed target of miR-32-5p. In this study, we confirmed the existence of GAS5/miR-32-5p/PTEN signaling pathway in pancreatic cancer cell metastasis. As the specific regulation mechanism between lncRNA and miRNA is complex, further work is needed to clarify the precise interactions between GAS5 and miR-32-5p, which is also the focus of our subsequent research.

PTEN is a well-known tumor suppressor with lipid phosphatase activity in a number of human cancers, such as endometrial cancer and lung cancer [20, 27]. PTEN suppression by miR-32 was conducived to the proliferation and invasion of colorectal cancer cells. In the present study, PTEN was indicated to down-regulate in PC tissues and cells, and could be elevated by GAS5 via inhibiting miR-32-5p. PTEN could block PI3K/Akt signaling pathway activation, resulting in the inhibition on the proliferation and survival of PC cells [16]. The present study showed that GAS5 suppressed the proliferation, migration and invasion of PC cells through regulating miR-32-5p/PTEN axis. Herein, it was concluded that the adverse effect of GAS5 on PC metastasis was, at least in part, mediated by the PTEN-induced tumor-suppressor pathway. This conclusion was further supported the results of in vivo experiments. GAS5 overexpression significantly abrogated PC cells tumorigenesis in vivo accompanied by decreased miR-32-5p and increased PTEN.

## Conclusion

In summary, the aim of the present study was to reveal the role and underlying mechanism of lncRNA GAS5 in PC cell metastasis. The main new achievement of this study includes: (1) LncRNA GAS5 inhibited the migration and invasion of PC cells; (2) miR-32-5p was increased in PC tissues and cells, and was associated with the migration and invasion of PC cells; (3) we further explicitly confirmed the existence of GAS5/miR-32-5p/PTEN signaling pathway in pancreatic cancer cell metastasis. These results may have contributed the development of GAS5-based therapeutic approaches for PC.

## Additional files

**Additional file 1: Table S1.** The characteristics of patients.

**Additional file 2: Figure S1.** GAS5 regulated the expression of PTEN through miR-32-5p. PC cells PANC-1 and BxPC-3 were transfected with miR-32-5p inhibitor plus si-control or miR-32-5p inhibitor plus si-GAS5. **a**–**b** The expression of PTEN was determined both at mRNA and protein level.

**Additional file 3: Figure S2.** GAS5 regulated the viability, migration and invasion of PC cells through miR-32-5p. PC cells PANC-1 and BxPC-3 were transfected with miR-32-5p inhibitor plus si-control or miR-32-5p inhibitor plus si-GAS5. The detection of **a** the relative cell viability, **b** migration and **c** invasion of PC cells treated as indicated.
