# Supplementary material for: Long non-coding RNA GAS5 suppresses pancreatic cancer metastasis through modulating miR-32-5p/PTEN axis
Source: Cell Biosci. 2017 Dec 4;7:66. doi: 10.1186/s13578-017-0192-0 (PMC5715988; doi:10.1186/s13578-017-0192-0)
Supplement: Supplementary file 1 — Additional file 1: Table S1. The characteristics of patients. [file 13578_2017_192_MOESM1_ESM.doc]

Table S1

| **Parameter** | **n** |
| --- | --- |
| **Gender** |  |
| Male | 13 |
| Female | 9 |
| **Age** (years)  mean (range) | 60.6 (49-80) |
| **Tumor location** |  |
| Pancreas head | 16 |
| Pancreas body | 4 |
| Pancreas tail | 2 |
| **Cancer grade** |  |
| 1 | 8 |
| 2 & 3 | 14 |
